# No role of the third-trimester inflammatory factors in the association of gestational diabetes mellitus with postpartum cardiometabolic indicators

**DOI:** 10.1186/s12884-024-06563-3

**Published:** 2024-05-15

**Authors:** Xiayan Yu, Wenjing Qiang, Kexin Gong, Yidan Cao, Shuangqin Yan, Guopeng Gao, Fangbiao Tao, Beibei Zhu

**Affiliations:** 1 Department of Maternal, Child and Adolescent Health, School of Public Health, Anhui Medical University, No 81 Meishan Road, Hefei, Anhui 230032 China; 2 Key Laboratory of Population Health Across Life Cycle (Anhui Medical University), Ministry of Education of the People’s Republic of China, No 81 Meishan Road, Hefei, Anhui 230032 China; 3 Anhui Provincial Key Laboratory of Environment and Population Health across the Life Course, Anhui Medical University, No 81 Meishan Road, Hefei, Anhui 230032 China; 4 NHC Key Laboratory of Study on Abnormal Gametes and Reproductive Tract, No 81 Meishan Road, Hefei, Anhui 230032 China; 5 Ma’anshan Maternal and Child Healthcare (MCH) Center, Ma’anshan, 243011 China

**Keywords:** Gestational diabetes mellitus, Cardiometabolic indicators, Inflammatory factors, Cohort study

## Abstract

**Background:**

The influence of gestational diabetes mellitus (GDM) on postpartum cardiometabolic indicators is primarily restricted to glucose and lipid metabolism, however the indicators for liver and kidney function have been rarely explored, and the role of the third-trimester inflammatory factors in these associations has never been investigated.

**Methods:**

Based on the Ma’anshan birth cohort (MABC), women with or without GDM history were selected and invited to participate in a 6-year postpartum follow-up. The fasting blood samples were collected to measure 16 comprehensive metabolic indicators during a 6-year postpartum follow-up: fasting plasma glucose (FPG), glycosylated hemoglobin (HbA1c), triglycerides (TG), total cholesterol (TC), uric acid (UA), blood urea nitrogen (BUN), serum creatinine (SCR), etc. Seven inflammatory factors, including TNF-α, IFN-γ, IL-1β, IL-6, IL-10, IL-12p70, and IL-17 A, were measured with serum samples collected during the third trimester of pregnancy. Linear regression models were used to analyze the associations between GDM and 6-year postpartum metabolic indicators, GDM and third-trimester inflammatory factors, and the third-trimester inflammatory factors and 6-year postpartum metabolic indicators. Mediating and moderating effect analyses were further performed to explore if the third-trimester inflammatory factors mediate or modify the association between GDM and postpartum cardiometabolic indicators.

**Results:**

From July 2021 to August 2022, 307 participants have been followed up, with 99 women with a prior GDM history. Compared with those without GDM, individuals with a prior history of GDM had significantly elevated levels of FPG (*β* = 0.40, 95% *CI*: 0.18 to 0.62, *P*_FDR_ < 0.001), HbA1c (*β* = 0.22, 95% *CI*: 0.09 to 0.34, *P*_FDR_ = 0.009), TyG (*β* = 0.22, 95% *CI*: 0.07 to 0.37, *P*_FDR_ = 0.024) at 6 years postpartum, and the association between GDM and SCR (*β* = 2.43, 95% *CI*: 0.02 to 4.85, *P*_FDR_ = 0.144) reached nominal significance level. GDM history was associated with a decreased level of third-trimester IL-17 A (*β* = -0.58, 95% *CI*: -0.99 to -0.18, *P*_FDR_ = 0.035). No significant association between third-trimester inflammatory factors and 6-year postpartum metabolic indicators was observed. And no mediating or moderating effect of third-trimester inflammatory factors was observed in those associations.

**Conclusion:**

A prior history of GDM was significantly associated with elevated FPG, HbA1c, and TyG in women at 6 years postpartum, whereas third-trimester inflammatory factors had no role in mediating or moderating these associations.

**Supplementary Information:**

The online version contains supplementary material available at 10.1186/s12884-024-06563-3.

## Background

Gestational diabetes mellitus (GDM) is defined as impaired glucose tolerance first diagnosed during pregnancy without a history of pre-existing diabetes [1], with a prevalence varying from 6.1 to 15.2% worldwide [2]. The adverse effects of GDM are not limited to short-term impacts such as macrosomia, premature delivery, and pre-eclampsia [3] but also increased lifetime risk of cardiovascular disease (CVD) [4, 5] in both mothers and their offspring. Of note, CVD is a leading cause of mortality and morbidity in women worldwide [6] and is also the case in China [7]. Therefore, for primary prevention of CVD, it is essential to establish the associations of prior GDM history with cardiovascular risk indicators and clarify the potential biological mechanisms.

It is well-established that metabolic indicators such as fasting plasma glucose (FPG), glycosylated hemoglobin (HbA1c), triglycerides (TG), total cholesterol (TC), and high-density lipoprotein cholesterol (HDL-C) are significantly associated with an increased risk of CVD in the general population [8–11]. Composite metabolic indicators like triglyceride-glucose index (TyG) and siMS (simple method for quantifying metabolic syndrome) index have also been used to predict the development of CVD [12, 13]. In recent years, many studies have reported associations between GDM and postpartum metabolic indicators, but the main focus of current studies has been on impaired glucose metabolism and dyslipidemia [14–16]. However, few studies have evaluated metabolic indicators of the liver and kidney, such as albumin (ALB), serum uric acid (UA), and blood urea nitrogen (BUN), which were significantly associated with the risk of CVD in the general population [17–19]. A meta-analysis on the CVD risk caused by GDM history showed that women with GDM had a 2-fold increased risk of developing CVD, irrespective of progression to T2DM [20]. Thus, for women with a prior GDM history, only an impaired glucose tolerance test postpartum might not be enough. Therefore, to gain a more comprehensive understanding of the mechanisms of developing CVD risk in GDM, it is essential to explore the relationship between GDM and cardiometabolic indicators using a comprehensive set of metabolic indicators.

The mechanisms underlying the doubled risk of CVD in women with previous GDM are unclear. Inflammatory factors play an important role in the development of both GDM and CVD. The occurrence of GDM may cause the persistence of chronic inflammation during pregnancy leading to changes in the number and type of immune cells and the release of pro-inflammatory factors [21]. Several clinical studies and animal experiments have shown that inflammatory factors influence the development of CVD by altering various signaling pathways that could promote the proliferation and migration of vascular smooth muscle cells (VSMC) and induce vascular endothelial dysfunction [22–25]. However, to our knowledge, no previous study has investigated the role of maternal inflammatory factors in linking GDM history with postpartum cardiovascular health indicators.

Therefore, based on the Ma’anshan birth cohort study (MABC), 99 women with GDM history and 208 women without were successfully followed at 6 years postpartum, with data from a comprehensive set of metabolic indicators and the third-trimester inflammatory factors, our study aims first to clarify the associations between GDM history and postpartum cardiometabolic indicators and second to examine the role of maternal inflammatory factors in them.

## Methods

### Study population

To investigate the long-term impact of GDM on women’s health postpartum and the modifiable factors, we randomly selected a 1:2 ratio of 120 participants with prior GDM history and 240 women without it from the Ma’anshan birth cohort (MABC). MABC was a population-based prospective cohort established in Ma’anshan Maternal and Child Health Care Center, which aims to investigate the effects of maternal environmental exposures on health outcomes of children’s development. The details of MABC’s inclusion and exclusion criteria can be found elsewhere [26]. From May 2013 to September 2014, 3474 pregnant women were recruited at their first visit, and their information and fasting blood samples were collected during the first, second, and third trimesters of pregnancy. The blood samples were centrifuged and stored in the − 80℃ refrigerator for future detection. Of them, women with accurate GDM diagnostic information were eligible for further follow-up. The study obtained ethics approval from the ethics committee of Anhui Medical University (20131195, 20210732). Written informed consent was obtained from all participating women.

### Simple size

Comparing women without GDM, the between-group effect size (Cohen’s d) of CVD postpartum proportion was assumed to be at least 0.38, which was demonstrated in a previous study [20]. Based on a dropout rate of 20% and an allocation rate of 1: 2 (GDM group versus non-GDM group), which was decided by the incidence of GDM in the original cohort and financial resources, the sample size of 103 women in the GDM group and 205 women in the non-GDM group was calculated using PASS version 21.0.3 (NCSS, LLC. Kaysville, Utah, USA), with an α of 5% and a statistical power (1-β) of 80%. Besides, considering the likelihood of GDM during follow-up in participants who were pre-determined to be in the non-GDM group, the final sample size of 120 women in the GDM and 240 women in the non-GDM group was proposed. And we selected 360 participants from women with or without prior GDM history in the cohort using a computerized random number method.

### Measuring tools

#### Sociodemographic information

Information on demographic characteristics (age, ethnics, education, income, etc.) and lifestyle was collected through self-administered questionnaires at 6-year postpartum follow-up. Information during pregnancy was retrieved from the baseline database.

#### Lifestyle information

The lifestyle assessments of sleeping, physical activity, and dietary were performed during 6-year postpartum follow-up:

A modified Chinese version of the Pittsburgh Sleep Quality Index (PSQI) scale, a validated and wildly used tool to measure sleep quality, was used to evaluate sleep quality [27]. It includes a total of 14 self-rated items, of which each has a range of 0–3 points [28]. The total PSQI score could be classified into 3 categories: excellent, average, and poor.

The International Physical Activity Questionnaire (IPAQ), with good reliability and validity [29], was used to assess the physical activity of the participants over the past 7 days, and the results were classified as low, moderate, and high physical activity levels.

The Food Frequency Questionaire (FFQ) investigated the frequency (daily, weekly, monthly, and yearly), quantity, and grams of food intake, which was used to evaluate the dietary habits and dietary nutrition of participants in the past year as red meat intake was strongly associated with the risk of CVD [30].

#### Anthropometric parameters

The height and weight of individuals were recorded to the nearest 0.1 kg and 0.1 cm, using a human body composition analyzer (GAIA KIKO, JAWON, Seoul, South Korea). Waist circumference (WC) at the level of the umbilicus was measured by a trained professional using non-stretchable sprung tape with the participants in the resting-standing position. Blood pressure was measured by one trained researcher using a mercury sphygmomanometer, and the average of the two readings was taken as the individual’s blood pressure value.

#### Diagnosis of GDM

During the 24 ∼ 28 week of pregnancy, women were given 75 g of glucose to consume orally for the OGTT at the center. The OGTT was conducted in the morning after a minimum of 8 h of fasting overnight. GDM was diagnosed if the plasma glucose levels met or exceeded either of the following values: 5.1 mmol/L for fasting glucose, 10.0 mmol/L at 1 h, and 8.5 mmol/L at 2 h [31].

### Assessment of inflammatory factors and metabolic indicators

Seven inflammatory factors, including TNF-α, IFN-γ, IL-1β, IL-6, IL-10, IL-12p70, IL-17 A, in the serum of participants from the third-trimester were measured by multi-bead enzyme-free analyzer (MILLIPLEX® MAP, Merck Millipore, Germany) using customized Human High Sensitivity Serum Factor Kit (MilliporeMAT Kit, Cat. No. HSTCMAG-28SK) between December 2018 and October 2019 [32]. The data were analyzed by Milliplex Analyst 5.1.

Participants’ fasting blood was collected by trained staff during a 6-year postpartum follow-up. The metabolic indicators were detected by the automatic biochemical analyzer (BECKMAN LX20, Beckman Coulter, Inc., USA), including fasting plasma glucose (FPG), glycosylated hemoglobin (HbA1c), hemoglobin F (HbF), insulin (RI), triglycerides (TG), total cholesterol (TC), high-density lipoprotein (HDL), Low-density lipoprotein (LDL), total protein (TP), albumin (ALB), apolipoprotein A (ApoA), apolipoprotein B (ApoB), uric acid (UA), blood urea nitrogen (BUN), serum creatinine (SCR) and cystatin (CYS).

Abnormal FPG was defined as FPG ≧ 6.1 mmol/L and abnormal HbA1c was defined as HbA1c ≧ 6.5% [33]. Dyslipidemia was divided into 4 categories [34]: Hypercholesterolemia (TC ≧ 5.2 mmol/L); Hypertriglyceridemia (TG ≧ 1.7 mmol/L); Hypo-HDLemia (HDL ≦ 5.2 mmol/L); Hyper-LDLemia (LDL ≧ 3.4 mmol/L). Metabolic syndrome (MetS) was diagnosed if 3 or more of the following 5 items were met [33]: (1) central obesity with WC (female) ≧ 85 cm; (2) FPG ≧ 6.1 mmol/L or 2 h PG ≧ 7.8 mmol/L or confirmed DM; (3) SBP/DBP ≧ 130/85 mmHg or confirmed hypertension; (4) fasting TG ≧ 1.70mmol/L; and (5) fasting HDL-C < 1.04mmol/L (female).

### Calculation of integrated metabolic index

Two integrated metabolic indices, the triglyceride-glucose (TyG) index and the Simple Method for Quantifying Metabolic Syndrome (siMS) score, were calculated. The TyG index was calculated from the levels of TG and FPG detected 6 years postpartum [35] with the following formula:

$${\text{TyG}} = {\text{ln}}\,{\text{(}}\frac{{{\text{TG}} \times {\text{FPG}}}}{2})$$

The siMS score was calculated from WC, height, FPG, TG, systolic blood pressure (SBP), and HDL measured 6 years postpartum [36], which was calculated as follows:

$$\text{s}\text{i}\text{M}\text{S} \text{s}\text{c}\text{o}\text{r}\text{e}=\frac{2\times \text{W}\text{C}}{\text{h}\text{e}\text{i}\text{g}\text{h}\text{t}}+\frac{\text{F}\text{P}\text{G}}{5.6}+\frac{\text{T}\text{G}}{1.7}+\frac{\text{S}\text{B}\text{P}}{130}-\frac{\text{H}\text{D}\text{L}}{1.28}$$

### Statistical analysis

Categorical variables are represented by frequencies and percentages, and continuous data are expressed as the mean ± standard deviation. The third-trimester inflammatory factors were log-transformed with a base of 2, so that the transformed distribution approximated a normal distribution for subsequent analysis [37]. If the concentrations of inflammatory factors were below the limit of detection (LOD), LOD / $\sqrt{2}$ was used instead. First, the chi-square test and the t-test were used to evaluate differences in characteristics between the GDM and non-GDM groups. Adjusted covariates were selected based on biological plausibility and the results of t-tests and chi-square tests, of which *p*-values less than 0.1 were included. Second, linear regression was used to determine the association between GDM and metabolic indicators at 6 years postpartum, and binary logistics was used to analyze the association between GDM and the risks of metabolic abnormalities at 6 years postpartum. Besides linear regression was also used to determine the association between GDM and seven third-trimester inflammatory factors, and the association between third-trimester inflammatory factors and metabolic indicators at 6 years postpartum. Based on the results, we use the PROCESS 3.3 plugin to analyze the moderating and mediating effect of third-trimester inflammatory factors in the association between GDM and significant metabolic indicators.

To account for multiple testing, two-sided *p* values were adjusted according to the Benjamini/Hochberg (B/H) method to control the false discovery rate (FDR). A statistically significant association was determined if its corresponding B/H-adjusted *p* value was less than 0.05, corresponding to an FDR of 5%. Risks are described as unadjusted and adjusted Relative risks (*RRs*) with 95% confidence intervals (*CI*s). The adjusted RRs and its 95% *CI*s were transformed from adjusted odd ratios (ORs) and 95% *CI*s calculated by binary logistic regression model using the formula from previous study [38]. All tests were two-sided, and *p*-values below 0.05 indicated significance. All statistical analyses were performed using SPSS version 23.0.

## Results

**Fig. 1:**
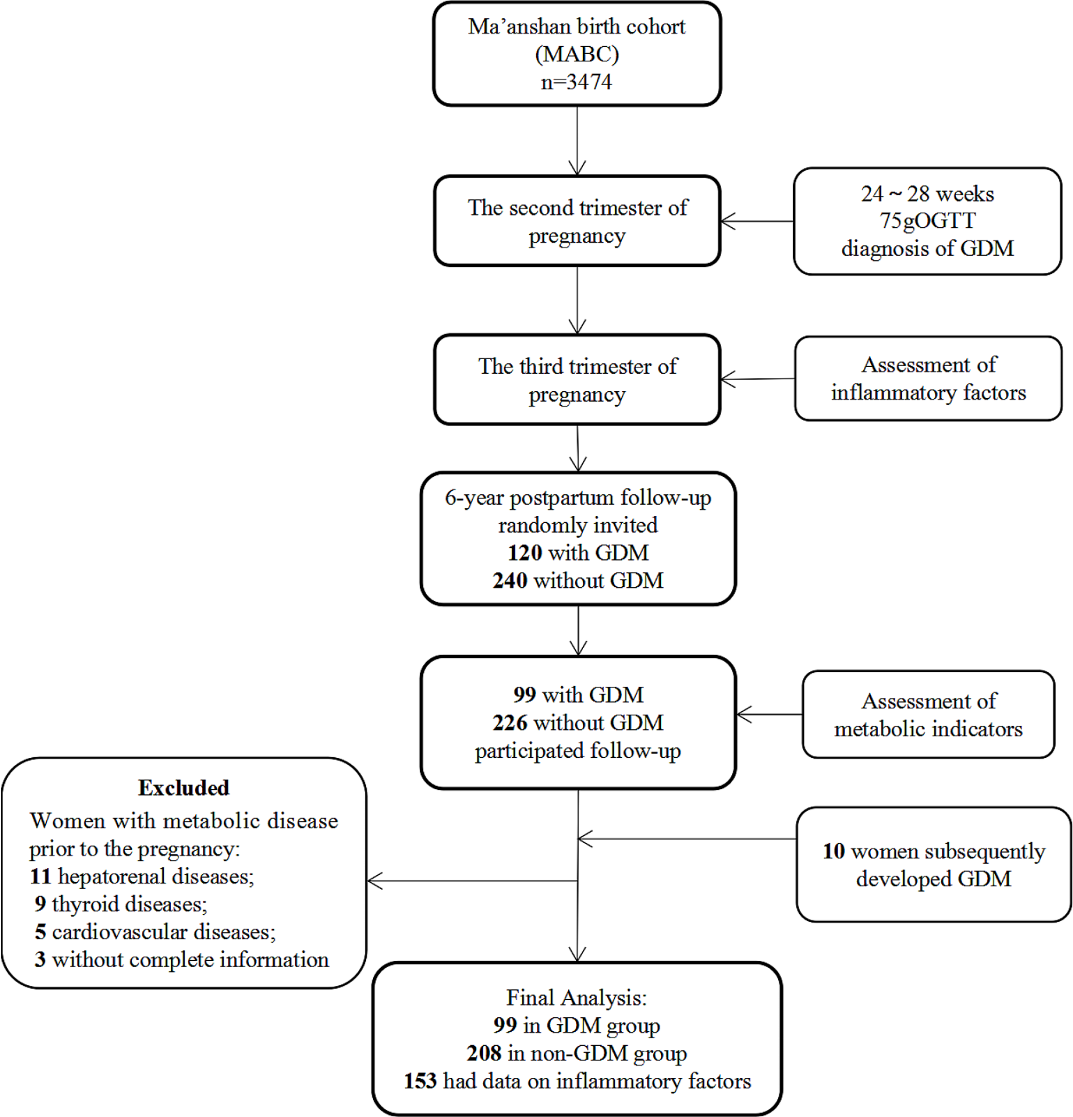
The flowchart of study

From July 2021 to August 2022, of 360 women who received our invitations for follow-up, 335 agreed to participate and have been successfully followed. Since 10 women without GDM in the index pregnancy developed GDM during subsequent pregnancies, these 10 women were transferred to the GDM group. After excluding women with pre-pregnancy hepatorenal diseases (*n* = 11), thyroid diseases (*n* = 9), cardiovascular diseases (*n* = 5), and 3 without complete information, our current study included a final 99 women in the GDM group and 208 women in the non-GDM group. The flowchart is shown in Fig. 1.

Table 1 shows the 6-year postpartum characteristics of the participants, comparing those with prior GDM history and those without it. Compared with women without GDM, those with prior GDM were older, had higher pre-pregnancy BMI and current BMI, and had a higher proportion of DM family history.

**Table 1:** Comparison of 6-year postpartum characteristics between the GDM and Non-GDM groups

Characteristics	Total sample (*n* = 307)	Non-GDM (*n* = 208)	GDM (*n* = 99)	*P*
Age (years), mean (SD)	35.82 (4.11)	35.15 (3.88)	37.22 (4.27)	**< 0.001**
Pre-pregnancy BMI, mean (SD)	21.04 (3.19)	20.52 (2.89)	22.14 (3.52)	**< 0.001**
Current BMI, mean (SD)	23.38 (3.73)	22.77 (3.49)	24.63 (4.02)	**< 0.001**
Gravidity, n (%)				0.718
1	95 (30.9)	63 (30.3)	32 (32.3)	
≥ 2	212 (69.1)	145 (69.7)	67 (67.7)	
Parity, n (%)				0.216
1	207 (67.4)	145 (69.7)	62 (62.6)	
≥ 2	100 (32.6)	63 (30.3)	37 (37.4)	
Marital status, n (%)				0.141
Married	293 (95.4)	196 (94.2)	97 (98.0)	
Unmarried or others	14 (4.6)	12 (5.8)	2 (2.0)	
Income (10k CNY/year), n (%)				0.553
< 5	45 (14.7)	31 (14.9)	14 (14.1)	
5 ∼ 9.99	118 (38.4)	76 (36.5)	42 (42.4)	
10 ∼ 19.99	97 (31.6)	65 (31.3)	32 (32.3)	
20 ∼ 29.99	27 (8.8)	22 (10.6)	5 (5.1)	
≥ 30	20 (6.5)	14 (6.7)	6 (6.1)	
Education, n (%)				0.628
Middle school / below	42 (13.7)	26 (12.5)	16 (16.2)	
High school	110 (35.8)	74 (35.6)	36 (36.4)	
Junior college / above	155 (50.5)	108 (51.9)	47 (47.4)	
Employment situation, n (%)				0.588
Unemployed	108 (35.2)	70 (33.7)	38 (38.4)	
Mental labor	165 (53.7)	116 (55.8)	49 (49.5)	
Manual labor	34 (11.1)	22 (10.6)	12 (12.1)	
Abnormal childbearing history, n (%)				0.475
Yes	38 (12.4)	24 (11.5)	14 (14.1)	
No	269 (87.6)	184 (99.5)	85 (85.9)	
DM family history, n (%) ^a^				**< 0.001**
Yes	102 (36.4)	57 (29.4)	45 (52.3)	
No	178 (63.6)	137 (70.6)	41 (47.7)	
CVD family history, n (%) ^b^				0.183
Yes	81 (30.9)	52 (28.4)	29 (36.7)	
No	181 (69.1)	131 (71.6)	50 (63.3)	
Drinking history, n (%)				0.328
Yes	92 (30.0)	66 (31.7)	26 (28.3)	
No	215 (70.0)	142 (68.3)	73 (73.7)	
Smoking history, n (%)				0.624
Yes	13 (4.2)	8 (3.8)	5 (5.1)	
No	294 (95.8)	200 (96.2)	94 (94.9)	
Classification of physical activity, n (%) ^c^				0.667
Low	56 (18.5)	37 (18.0)	19 (19.6)	
Moderate	117 (38.6)	77 (37.4)	40 (41.2)	
High	130 (42.9)	92 (44.7)	38 (39.2)	
Classification of sleep quality, n (%)				0.137
Excellent	178 (58.0)	127 (61.1)	51 (51.5)	
Average	119 (38.8)	73 (35.1)	46 (46.5)	
Poor	10 (3.3)	8 (3.8)	2 (2.0)	
Red meat intake (grams/day), mean (SD)	75.52 (103.23)	64.76 (52.15)	98.14(163.61)	0.050

^a^: 27 cases with unclear DM family history were treated as missing values;

^b^: 45 cases with unclear CVD family history were treated as missing values.

^c^: 6 cases with unclear physical activity were treated as missing values.

Table 2 shows the association between GDM history and metabolic indicators at 6 years postpartum. After adjusting for age, pre-pregnant BMI, education, income, parity, DM family history, CVD family history in model 1, compared with women without GDM, those with prior GDM had significantly higher levels of FPG (*β* = 0.39, 95% *CI*: 0.18 to 0.61, *P*_FDR_<0.001), HbA1c (*β* = 0.22, 95% *CI*: 0.10 to 0.34, *P*_FDR_<0.001), and TyG (*β* = 0.23, 95% *CI*: 0.08 to 0.38, *P*_FDR_ = 0.012) at 6 years postpartum with corrected *P*_FDR_ below 0.05. While the association between GDM history and TG (*β* = 0.22, 95% *CI*: 0.02 to 0.43, *P*_FDR_ = 0.110), HbF (*β* = 0.07, 95% *CI*: 0.00 to 0.14, *P*_FDR_ = 0.110), RI (*β* = 1.81, 95% *CI*: 0.05 to 3.57, *P*_FDR_ = 0.110), ApoA (*β* = 0.05, 95% *CI*: 0.00 to 0.10, *P*_FDR_ = 0.110), and siMS (*β* = 0.23, 95% *CI*: 0.03 to 0.42, *P*_FDR_ = 0.104) only reached nominal significance level. Based on Model 1, Model 2 further adjusted for sleeping quality, physical activity, and red meat consumption, the association between GDM history and 6-year postpartum FPG (*β* = 0.40, 95% *CI*: 0.18 to 0.62, *P*_FDR_<0.001), HbA1c (*β* = 0.22, 95% *CI*: 0.09 to 0.34, *P*_FDR_ = 0.009), TyG (*β* = 0.22, 95% *CI*: 0.07 to 0.37, *P*_FDR_ = 0.024) remained significant. Still, the association between GDM history and ApoA (*β* = 0.06, 95% *CI*: 0.01 to 0.12, *P*_FDR_ = 0.090), SCR (*β* = 2.43, 95% *CI*: 0.02 to 4.85, *P*_FDR_ = 0. 144), siMS (*β* = 0.20, 95% *CI*: 0.01 to 0.40, *P*_FDR_ = 0.144) only reached nominal significance level.

**Table 2:** Association between GDM history and 6-year postpartum metabolic indicators

Metabolic indicators	Non-GDM (*n* = 208)	GDM (*n* = 99)	Model 1^a^			Model 2^b^		
			*β* (95%*CI*)	*P*_unjusted_	*P*_FDR_	*β* (95%*CI*)	*P*_unjusted_	*P*_FDR_
FPG (mmol/L)	4.69 (0.73)	5.40 (1.52)	0.39 (0.18 to 0.61)	**<0.001**	**<0.001**	0.40 (0.18 to 0.62)	**<0.001**	**<0.001**
TC (mmol/L)	4.27 (0.88)	4.52 (0.78)	0.10 (-0.15 to 0.34)	0.430	0.595	0.12 (-0.13 to 0.37)	0.356	0.534
TG (mmol/L)	0.97 (0.54)	1.34 (0.98)	0.22 (0.02 to 0.43)	**0.035**	0.110	0.20 (-0.01 to 0.41)	0.068	0.175
HDL (mmol/L)	1.49 (0.30)	1.42 (0.33)	-0.02 (-0.10 to 0.07)	0.710	0.887	-0.01 (-0.09 to 0.08)	0.864	0.901
LDL (mmol/L)	2.52 (0.80)	2.71 (0.70)	-0.01 (-0.23 to 0.21)	0.959	0.959	0.01 (-0.21 to 0.24)	0.901	0.901
HbA1c (%)	5.26 (0.43)	5.68 (0.87)	0.22(0.10 to 0.34)	**<0.001**	**<0.001**	0.22 (0.09 to 0.34)	**0.001**	**0.009**
HbF (%)	0.41 (0.24)	0.46 (0.25)	0.07(0.00 to 0.14)	**0.049**	0.110	0.06 (-0.01 to 0.13)	0.082	0.182
RI (uU/mL)	10.17 (5.86)	12.91 (7.75)	1.81(0.05 to 3.57)	**0.044**	0.110	1.53 (-0.28 to 3.34)	0.098	0.182
TP (g/L)	72.85 (3.76)	73.75 (3.89)	0.68(-0.35 to 1.72)	0.195	0.319	0.55 (-0.51 to 1.61)	0.304	0.497
ALB (g/L)	46.70 (2.35)	46.68 (2.26)	0.11(-0.54 to 0.77)	0.739	0.887	0.34 (-0.43 to 0.91)	0.484	0.622
ApoA (g/L)	1.12 (0.19)	1.14 (0.21)	0.05(0.00 to 0.10)	**0.048**	0.110	0.06 (0.01 to 0.12)	**0.020**	0.090
ApoB (g/L)	0.71 (0.17)	0.77 (0.16)	0.01(-0.04 to 0.05)	0.820	0.923	0.01 (-0.04 to 0.06)	0.759	0.901
BUN (umol/L)	4.59 (1.05)	4.60 (1.10)	0.02(-0.28 to 0.32)	0.887	0.939	0.02 (-0.29 to 0.33)	0.890	0.901
SCR (umol/L)	56.97 (7.85)	59.08 (9.36)	2.00(-0.33 to 4.34)	0.093	0.167	2.43 (0.02 to 4.85)	**0.048**	0.144
UA (umol/L)	270.70 (67.75)	292.53 (74.79)	19.07(-0.83 to 38.98)	0.060	0.120	16.89 (-3.29 to 37.06)	0.101	0.182
CYS (mg/L)	0.71 (0.12)	0.71 (0.13)	-0.02(-0.05 to 0.01)	0.257	0.386	-0.01 (-0.05 to 0.02)	0.404	0.559
siMS	1.98 (0.65)	2.46 (0.93)	0.23 (0.03 to 0.42)	**0.023**	0.104	0.20 (0.01 to 0.40)	**0.045**	0.144
TyG	8.08 (0.52)	8.47 (0.62)	0.23(0.08 to 0.38)	**0.002**	**0.012**	0.22 (0.07 to 0.37)	**0.004**	**0.024**

Continuous variables are expressed as mean (standard deviation),

^a^:model 1 adjusted for age, pre-pregnant BMI, education, income, parity, DM family history, CVD family history;

^b^:Model 2 adjusted for age, pre-pregnant BMI, education, income, parity, DM family history, CVD family history, sleeping quality, physical activity, red meat consumption.

Table 3 shows the associations between GDM history and metabolic abnormalities at 6 years postpartum. Adjusted model 1 revealed that the association between GDM history and FPG abnormality (*RR* = 6.95, 95% *CI*: 1.34 to 29.48, *P*_FDR_ = 0.077), hypo-HDLemia (*RR* = 6.58, 95% *CI*: 1.52 to 20.07, *P*_FDR_ = 0.077) only reached nominal significance level. Model 2 also showed that the association between GDM history and FPG abnormality (*RR* = 7.68, 95% *CI*: 1.33 to 34.25, *P*_FDR_ = 0.081), MetS (*RR* = 4.46, 95% *CI*: 1.01 to 15.21, *P*_FDR_ = 0.112), hypo-HDLemia (*RR* = 5.94, 95% *CI*: 1.37 to 18.71, *P*_FDR_ = 0.081) only reached nominal significance.

Of the 307 participants included in the analysis, 153 had data on inflammatory factors in the third trimester of pregnancy, and 39 of these had a prior history of GDM. As shown in Table 4, after adjusting for age, pre-pregnant BMI, education, income, parity, DM family history, and CVD family history, a significant inverse association was observed between GDM and the third-trimester level of IL-17 A (*β* = -0.58, 95% *CI*: -0.99 to -0.18, *P*_FDR_ = 0.035) and an inverse association between GDM and IFN-γ (*β* = -0.46, 95% *CI*: -0.85 to -0.07, *P*_FDR_ = 0.07) was observed, but the corrected *P* value only reached a nominal significance level.

Table 5 shows the association between inflammatory factors in the third trimester and metabolic indicators at 6 years postpartum. No statistically significant association was found between any of the inflammatory factors and 6-year postpartum metabolic indicators.

Based on the above results, since GDM was significantly associated with 6-year postpartum FPG, HbA1c, and TyG, we only presented the results of the mediating and moderating effect analysis of the third-trimester inflammatory factors in linking these associations in eTable 1 and eTable 2 (Supplement). Still, neither of the seven inflammatory factors was observed to have any mediating or moderating effect.

**Table 3:** Associations between GDM history and 6-year postpartum metabolic abnormalities

Metabolic abnormalities	Non-GDM (*n* = 208)	GDM (*n* = 99)	Model 1^a^			Model 2^b^		
			*RR* (95%*CI*)	*P*_unjusted_	*P*_FDR_	*RR* (95%*CI*)	*P*_unjusted_	*P*_FDR_
Classification of FPG				**0.022**	0.077		**0.023**	0.081
Normal	206 (99.0)	85 (85.9)	1.00			1.00		
abnormal	2 (1.0)	14 (14.1)	6.95 (1.34 to 29.48)			7.68 (1.33 to 34.25)		
Classification of HbA1c				0.100	0.175		0.235	0.274
Normal	207 (99.5)	93 (93.9)	1.00			1.00		
abnormal	1 (0.5)	6 (6.1)	16.93 (0.57 to 153.93)			95.06 (0.04 to 207.95)		
MetS				0.076	0.175		**0.048**	0.112
No	203 (97.6)	85 (85.9)	1.00			1.00		
Yes	5 (2.4)	14 (14.1)	3.60 (0.87 to 12.29)			4.46 (1.01 to 15.21)		
Hypercholesterolemia				0.304	0.355		0.210	0.274
No	189 (90.9)	81 (81.8)	1.00			1.00		
Yes	19 (9.1)	18 (18.2)	1.48 (0.69 to 2.93)			1.63 (0.75 to 3.22)		
Hypertriglyceridemia				0.257	0.355		0.230	0.274
No	190 (91.3)	77 (77.8)	1.00			1.00		
Yes	18 (8.7)	22 (22.2)	1.55 (0.72 to 3.10)			1.60 (0.74 to 3.20)		
Hypo-HDLemia				**0.013**	0.077		**0.019**	0.081
No	203 (97.6)	90 (90.9)	1.00			1.00		
Yes	5 (2.4)	9 (9.1)	6.58 (1.52 to 20.07)			5.94 (1.37 to 18.71)		
Hyper-LDLemia				0.806	0.806		0.935	0.935
No	192 (92.3)	86 (86.9)	1.00			1.00		
Yes	16 (7.7)	13 (13.1)	0.89 (0.34 to 2.17)			0.96 (0.37 to 2.36)		

Categorical variables are expressed as number of cases (ratio),

^a^:model 1 adjusted for age, pre-pregnant BMI, education, income, parity, DM family history, CVD family history;

^b^:Model 2 adjusted for adjusted for age, pre-pregnant BMI, education, income, parity, DM family history, CVD family history, sleeping quality, physical activity, red meat consumption.

**Table 4:** Association between GDM history and the third-trimester inflammatory factors

Inflammatory factors	Non-GDM (*n* = 114)	GDM (*n* = 39)	Model 1^a^			Model 2^b^		
			*β* (95% *CI*)	*P*_unjusted_	*P*_FDR_	*β* (95% *CI*)	*P*_unjusted_	*P*_FDR_
IFN-γ	6.76 (4.29, 11.77)	4.99 (3.47, 9.35)	-0.33 (-0.66 to 0.00)	0.050	0.175	-0.46 (-0.85 to -0.07)	**0.020**	0.07
IL-1B	1.07 (0.78, 1.56)	0.91 (0.67, 1.67)	-0.13 (-0.39 to 0.14)	0.340	0.567	-0.23 (-0.54 to 0.08)	0.137	0.243
IL-6	3.06 (2.29, 4.93)	2.93 (1.65, 6.67)	0.18 (-0.25 to 0.60)	0.405	0.567	0.16 (-0.35 to 0.67)	0.530	0.618
IL-10	12.24 (8.43, 16.66)	14.24 (7.25, 20.14)	-0.03 (-0.37 to 0.31)	0.867	0.867	-0.23 (-0.65 to 0.19)	0.273	0.382
IL-12	2.47 (1.75, 4.26)	1.98 (1.42, 3.25)	-0.22 (-0.54 to 0.10)	0.182	0.425	-0.28 (-0.66 to 0.09)	0.139	0.243
IL-17 A	8.29 (4.74, 14.09)	5.73 (3.86, 9.04)	-0.39 (-0.74 to -0.05)	**0.027**	0.175	-0.58 (-0.99 to -0.18)	**0.005**	**0.035**
TNF-α	5.22 (3.98, 8.49)	6.00 (3.65, 10.10)	0.06 (-0.24 to 0.36)	0.700	0.817	0.03 (-0.33 to 0.39)	0.866	0.866

Concentration of inflammatory factors are expressed as median (interquartile range);

^a^:model 1 was unadjusted;

^b^:Model 2 adjusted for age, pre-pregnant BMI, education, income, parity, DM family history, CVD family history.

**Table 5:** Association between third-trimester inflammatory factors and 6-year postpartum metabolic indicators

Metabolic indicators	*β* (95% *CI*)						
	IFN-γ	IL-1B	IL-6	IL-10	IL-12	IL-17 A	TNF-α
FPG	0.09 (-0.01 to 0.18)	0.00 (-0.12 to 0.12)	-0.01 (-0.08 to 0.06)	0.07 (-0.02 to 0.16)	0.02 (-0.08 to 0.11)	0.06 (-0.03 to 0.14)	0.04 (-0.07 to 0.14)
TC	0.01 (-0.17 to 0.19)	0.07 (-0.16 to 0.29)	0.08 (-0.06 to 0.22)	0.00 (-0.17 to 0.17)	0.01 (-0.17 to 0.19)	0.02 (-0.15 to 0.18)	0.04 (-0.15 to 0.24)
TG	0.06 (-0.08 to 0.21)	0.11 (-0.08 to 0.30)	0.04 (-0.07 to 0.16)	0.12 (-0.02 to 0.26)	0.09 (-0.07 to 0.24)	0.10 (-0.04 to 0.24)	0.08 (-0.09 to 0.24)
HDL	0.01 (-0.04 to 0.06)	0.01 (-0.05 to 0.08)	0.00 (-0.04 to 0.04)	0.05 (0.00 to 0.09)	0.00 (-0.05 to 0.06)	-0.01 (-0.06 to 0.04)	-0.02 (-0.08 to 0.03)
LDL	-0.02 (-0.18 to 0.15)	0.01 (-0.20 to 0.21)	0.03 (-0.10 to 0.16)	-0.08 (-0.23 to 0.07)	-0.02 (-0.19 to 0.15)	-0.01 (-0.16 to 0.14)	0.02 (-0.16 to 0.20)
HbA1c	-0.02 (-0.06 to 0.03)	-0.04 (-0.10 to 0.02)	-0.01 (-0.05 to 0.02)	0.01 (-0.04 to 0.05)	-0.03 (-0.08 to 0.01)	-0.02 (-0.07 to 0.02)	-0.02 (-0.07 to 0.03)
HbF	0.00 (-0.05 to 0.05)	-0.02 (-0.08 to 0.04)	0.00 (-0.03 to 0.04)	0.01 (-0.04 to 0.05)	0.00 (-0.05 to 0.05)	0.00 (-0.05 to 0.05)	0.00 (-0.06 to 0.05)
RI	0.47 (-0.67 to 1.61)	-0.17 (-1.62 to 1.28)	-0.07 (-0.97 to 0.82)	0.29 (-0.79 to 1.37)	0.73 (-0.45 to 1.91)	0.84 (-0.24 to 1.91)	1.24 (0.00 to 2.49)
TP	0.43 (-0.21 to 1.06)	0.38 (-1.18 to 0.43)	-0.28 (-0.77 to 0.22)	-0.49 (-1.09 to 0.11)	0.21 (-0.45 to 0.87)	-0.08 (-0.69 to 0.52)	0.53 (-0.17 to 1.23)
ALB	0.34 (-0.04 to 0.73)	0.12 (-0.37 to 0.61)	0.19 (-0.11 to 0.49)	-0.13 (-0.49 to 0.24)	0.17 (-0.24 to 0.57)	0.14 (-0.23 to 0.51)	0.37 (-0.05 to 0.80)
ApoA	0.02 (-0.02 to 0.05)	0.00 (-0.04 to 0.05)	0.00 (-0.02 to 0.03)	0.03 (0.00 to 0.06)	0.01 (-0.02 to 0.05)	0.00 (-0.04 to 0.03)	0.00 (-0.04 to 0.03)
ApoB	0.00 (-0.04 to 0.03)	-0.01 (-0.05 to 0.04)	0.00 (-0.02 to 0.03)	-0.01 (-0.05 to 0.02)	0.00 (-0.04 to 0.04)	0.00 (-0.04 to 0.03)	0.01 (-0.03 to 0.05)
BUN	0.02 (-0.17 to 0.21)	-0.09 (-0.33 to 0.15)	0.01 (-0.14 to 0.16)	-0.02 (-0.20 to 0.16)	0.01 (-0.19 to 0.20)	-0.03 (-0.21 to 0.15)	-0.11 (-0.32 to 0.10)
Scr	0.41 (-1.00 to 1.83)	0.62 (-1.17 to 2.41)	-0.06 (-1.16 to 1.05)	-0.06 (-1.40 to 1.28)	0.09 (-1.38 to 1.56)	0.36 (-0.98 to 1.70)	0.41 (-1.16 to 1.97)
UA	3.65 (-9.34 to 16.63)	2.75 (-13.71 to 19.21)	4.31 (-5.80 to 14.41)	5.50 (-6.74 to 17.74)	3.54 (-9.95 to 17.04)	2.38 (-9.93 to 14.69)	12.62 (-1.61 to 26.85)
Cys	-0.02 (-0.04 to 0.00)	-0.01 (-0.03 to0.02)	-0.01 (-0.03 to 0.01)	-0.01 (-0.03 to 0.01)	-0.02 (-0.04 to 0.00)	0.00 (-0.02 to 0.02)	-0.01 (-0.03 to 0.01)
siMS	0.05 (-0.08 to 0.18)	0.05 (-0.11 to 0.21)	0.02 (-0.08 to 0.12)	0.06 (-0.06 to 0.18)	0.07 (-0.07 to 0.20)	0.09 (-0.03 to 0.20)	0.08 (-0.06 to 0.22)
TyG	0.06 (-0.04 to 0.15)	0.02 (-0.10 to 0.13)	0.00 (-0.07 to 0.08)	0.07 (-0.02 to 0.16)	0.05 (-0.04 to 0.15)	0.06 (-0.03 to 0.15)	0.06 (-0.04 to 0.17)

The model adjusted for age, pre-pregnant BMI, education, income, parity, DM family history, CVD family history, sleeping quality, physical activity, red meat consumption.

## Discussion

Our study found that prior GDM history was significantly associated with 6-year postpartum metabolic indicators FPG, HbA1c, and TyG, indicating that GDM history could influence women’s glucose and lipid metabolism in early postpartum, emphasizing the importance of surveillance of those indicators. However, there was no significant effect of the third-trimester inflammatory factors in mediating or moderating these associations, suggesting novel mechanisms should be explored to understand how GDM history influences cardiovascular health.

Consistent with previous findings [15, 39–43], our study revealed that a prior history of GDM was associated with elevated levels of FPG, HbA1c, and the composite index TyG at 6 years postpartum. Regarding postpartum lipid metabolism indicators, previous studies and our study exhibited mixed results [44–48], with some finding positive associations [44–46] and others null associations [47, 48]. Explanations for the differences might firstly be attributed to the sample size, with small sample sizes in studies from Hungary and Natong [44, 46], and larger in studies from Iran and Louisiana [47, 48]. Secondly, the mentioned studies [44, 46] differed in the time point of follow-up, with some even having obvious between-group differences. For example, in the study of Hungary [44], the GDM and control groups were followed at (3.5 ± 0.6) and (8.2 ± 5.1) years (*P <* 0.001), respectively. The women in the study of Nantong [46] were followed at 1 year postpartum, however they were followed at over 7 years postpartum in Iranian and Louisiana studies [47, 48], and in our study were at over 6 years postpartum. Besides, both Hungary and Nantong studies missed important potential confounders such as sleep, physical activity, and dietary intake, which have been shown to have influences on lipid metabolism and risk of CVD [29, 49, 50]. Moreover, different GDM diagnostic criteria applied in different studies might be another explanation. Therefore, based on the existing studies, the association between GDM history and postpartum lipid metabolism could not be determined. Future studies with larger sample sizes, longer follow-up periods, and more comprehensive adjustments for confounding variables were needed.

Metabolic indicators of liver and kidney functions, which were influenced by endocrine disorders and could reflect the nutrition and protein metabolism, could be used to reflect the pathophysiological process of CVD [19]. Studies in the general population have shown that decreased ALB [17], increased UA [18] and BUN [19], which are indicators reflecting kidney function, were associated with an increased risk of CVD. Two studies [51, 52] from Poland and China showed among women with prior GDM, the levels of UA were positively associated with the risk of T2DM and prediabetes, which is also an important risk factor for CVD. Our study has shown that the original *P* value for SCR (*P* = 0.048), an indicator of kidney function, was significant. Even though the corrected *P* values of SCR (*P* = 0.048, *P*_FDR_ = 0.144) and ApoA (*P* = 0.020, *P*_FDR_ = 0.09) in our study were not significant after multiple testing corrections, the between-group differences in the levels of SCR and ApoA at 6 years postpartum still preliminarily hints that GDM might be associated with liver and kidney metabolic indicators postpartum, however the exact relationship remains to be further investigated.

To our surprise, our study found significantly lower levels of third-trimester IL-17 A in the GDM group than in the non-GDM group. There were no previous studies exploring the relationship between GDM and third-trimester IL-17 A, but a few descriptive studies have reported higher levels of other third-trimester inflammatory factors in the GDM groups [53, 54]. For example, a study in Hohhot, China, showed that IL-6 and IL-8 during pregnancy were higher in the GDM group (*n* = 60) compared with the non-GDM group (*n* = 60) [53], and an Indian study also showed differences in IL-6 and TNF-α in late pregnancy between the GDM group (*n* = 35) and the control group (*n* = 30) [54]. However, another study reported an insignificant association between GDM and third-trimester inflammatory factors, for example, a meta-analysis found TNF-α was slightly higher in the GDM group than in the control group but without significance [55]. Thus, due to the varied methods of measuring inflammatory factors and the small sample size of all existing studies, the incidence of GDM could impact the level of maternal inflammatory factors still needs further investigation.

Although pro-inflammatory factors such as TNF-α, IL-1, and IL-6 were found to contribute to the development of atherosclerosis by affecting vascular endothelial cell function, oxidized LDL, heat shock protein and HDL levels, etc., leading to the development of atherosclerosis [56], and were shown to participate in the glucose metabolism by involving in the insulin signaling pathways [57–59]. No association was found between any of the seven third-trimester inflammatory factors and metabolic indicators 6 years postpartum in our study. To our knowledge, no study has attempted to explore the association between maternal inflammatory factors and postpartum metabolic indicators in humans. Only animal experiments indicated that postnatal glucose metabolism alterations were associated with an inflammatory state during pregnancy in rats [60]. Accordingly, there is no moderating or mediating effect of the third-trimester inflammatory factors in any association between GDM and FPG, HbA1c, or TyG at 6 years postpartum either.

Our study has several strengths. First, a set of comprehensive metabolic indicators was examined, particularly for liver and renal functions, and two integrated metabolic indices, TyG and siMS score. Second, a range of lifestyle factors such as sleep, physical activity, and dietary intake were adjusted for, increasing the reliability of our results. More importantly, we innovatively explored the role of maternal inflammatory factors in these associations. However, some limitations should also be acknowledged. First, only seven third-trimester inflammatory factors were assayed, other important inflammatory indicators, such as IL-4 and C-reactive protein, were not included. Second, the long-term storage of serum samples might affect the accuracy of inflammatory factors assays, but all samples were stored under the same storage conditions and underwent a similar number of freeze-thaw cycles before being tested, the variability between samples was not altered. Third, the small sample size might affect the statistical power in detecting differences in this study. Fourth, we did not collect blood glucose indicators to objectively reflect glycemic control during late pregnancy, despite we have investigated treatments for GDM during pregnancy, and all participants reported only controlled dietary therapies. Fifth, we did not perform the OGTT to diagnose whether participants had developed T2DM during follow-up.

## Conclusion

A prior history of GDM was significantly associated with elevated FPG, HbA1c, and TyG at 6 years postpartum, whereas third-trimester inflammatory factors had no role in mediating or moderating these associations. Our findings remain to be further validated in a prospective study with a large sample size.

### Electronic supplementary material

Below is the link to the electronic supplementary material.

Supplementary Material 1

## Data Availability

Data are available from the corresponding author on reasonable request.

## Abbreviations

GDM: Gestational diabetes mellitus
MABC: Ma’anshan birth cohort
CVD: Cardiovascular disease
PSQI: The Pittsburgh sleep quality index
IPAQ: The international physical activity questionnaire
FFQ: The food frequency questionnaire
WC: Waists circumference
OGTT: Oral glucose tolerance test
FPG: Fasting plasma glucose
HbA1c: glycosylated hemoglobin
HbF: hemoglobin F
RI: Insulin
TG: Triglycerides
TC: Total cholesterol
HDL: High-density lipoprotein
LDL: Low-density lipoprotein
TP: Total protein
ALB: Albumin
ApoA: Apolipoprotein A
ApoB: Apolipoprotein B
UA: Uric acid
BUN: Blood urea nitrogen
SCR: Serum creatinine
CYS: Cystatin
MetS: Metabolic syndrome
TyG: triglyceride-glucose index
siMS: Quantifying metabolic syndrome score
LOD: The limit of detection
CI: Confidence internal
RR: Relative risk
