# Supplementary material for: No role of the third-trimester inflammatory factors in the association of gestational diabetes mellitus with postpartum cardiometabolic indicators
Source: BMC Pregnancy Childbirth. 2024 May 15;24:361. doi: 10.1186/s12884-024-06563-3 (PMC11095010; doi:10.1186/s12884-024-06563-3)
Supplement: Supplementary file 1 — Supplementary Material 1 [file 12884_2024_6563_MOESM1_ESM.docx]

**Supplemental Online Content**

**eTable 1.** The mediating effect of third-trimester inflammatory factors on the association between GDM and 6-year postpartum metabolic indicators

**eTable 2.** The moderating effect of third-trimester inflammatory factors on the association between GDM and 6-year postpartum metabolic indicators

This supplemental material has been provided by the authors to give readers additional information about their work.

**eTable 1.** The mediating effect of third-trimester inflammatory factors on the association between GDM and 6-year postpartum metabolic indicators

| Mediating variable | Total effect (95%*CI*) | Direct effect (95%*CI*) | Indirect effect (95%*CI*) |
| --- | --- | --- | --- |
| **FPG** |  |  |  |
| IFN-γ | 0.5903 (0.0000, 0.3814) | 0.6280 (0.0000, 0.4204) | -0.0377 (-0.1023, .0023) |
| IL-1B | 0.5903 (0.0000, 0.3814) | 0.5899 (0.0000, 0.3799) | 0.0004 (-0.0266, 0.0236) |
| IL-6 | 0.5903 (0.0000, 0.3814) | 0.6016 (0.0000, 0.3916) | -0.0114 (-0.0614, 0.0125) |
| IL-10 | 0.5903 (0.0000, 0.3814) | 0.5895 (0.0000, 0.3829) | 0.0008 (-0.0413, 0.0384) |
| IL-12 | 0.5903 (0.0000, 0.3814) | 0.5931 (0.0000, 0.3819) | -0.0028 (-0.0371, 0.0352) |
| IL-17A | 0.5903 (0.0000, 0.3814) | 0.6170 (0.0000, 0.4066) | -0.0267 (-0.0931, 0.0074) |
| TNF-α | 0.5903 (0.0000, 0.3814) | 0.5848 (0.0000, 0.3752) | 0.0055 (-0.0211, 0.0402) |
| **HbA1c** |  |  |  |
| IFN-γ | 0.3638 (0.0000, 0.2536) | 0.3681 (0.0000, 0.2565) | -0.0043 (-0.0256, 0.0137) |
| IL-1B | 0.3638 (0.0000, 0.2536) | 0.3604 (0.0000, 0.2501) | 0.0034 (-0.0081, 0.0228) |
| IL-6 | 0.3638 (0.0000, 0.2536) | 0.3712 (0.0000, 0.2606) | -0.0074 (-0.0299, 0.0087) |
| IL-10 | 0.3638 (0.0000, 0.2536) | 0.3635 (0.0000, 0.2539) | 0.0003 (-0.0195, 0.0150) |
| IL-12 | 0.3638 (0.0000, 0.2536) | 0.3586 (0.0000, 0.2474) | 0.0052 (-0.0102, 0.0269) |
| IL-17A | 0.3638 (0.0000, 0.2536) | 0.3619 (0.0000, 0.2500) | 0.0019 (-0.0171, 0.0223) |
| TNF-α | 0.3638 (0.0000, 0.2536) | 0.3649 (0.0000, 0.2541) | -0.0010 (-0.0171, 0.0108) |
| **TyG** |  |  |  |
| IFN-γ | 0.3001 (0.0026, 0.1064) | 0.3053 (0.0025, 0.1090) | -0.0052 (-0.0468, 0.0239) |
| IL-1B | 0.3001 (0.0026, 0.1064) | 0.2953(0.0031, 0.1011) | 0.0048 (-0.0197, 0.0398) |
| IL-6 | 0.3001 (0.0026, 0.1064) | 0.3151 (0.0016, 0.1211) | -0.0150 (-0.0707, 0.0156) |
| IL-10 | 0.3001 (0.0026, 0.1064) | 0.2996 (0.0026, 0.1066) | 0.0005 (-0.0286, 0.0321) |
| IL-12 | 0.3001 (0.0026, 0.1064) | 0.2963 (0.0033, 0.1005) | 0.0039 (-0.0291, 0.0365) |
| IL-17A | 0.3001 (0.0026, 0.1064) | 0.2992 (0.0031, 0.1024) | 0.0009 (-0.0476, 0.0410) |
| TNF-α | 0.3001 (0.0026, 0.1064) | 0.2987 (0.0029, 0.1039) | 0.0015 (-0.0244, 0.0235) |

**eTable 2.** The moderating effect of third-trimester inflammatory factors on the association between GDM and 6-year postpartum metabolic indicators

| **Variables** | **FPG** | |  | **HbA1c** | |  | **TyG** | |
| --- | --- | --- | --- | --- | --- | --- | --- | --- |
|  | ***β*** | ***t*** |  | ***β*** | ***t*** |  | ***β*** | ***t*** |
| GDM | 0.0681 | 0.222 |  | 0.1689 | 1.0181 |  | 0.2835 | 0.9666 |
| IFN-γ | 0.0688 | 1.2907 |  | -0.0040 | -0.1380 |  | 0.0144 | 0.2826 |
| GDM×IFN-γ | 0.2195 | 1.8441 |  | 0.0781 | 1.2775 |  | 0.0086 | 0.0792 |
| *R^2^* | 0.2235 | |  | 0.2297 | |  | 0.0593 | |
| *F* | 14.2938 | |  | 14.8140 | |  | 3.1302 | |
| GDM | 0.5930 | 5.5406 |  | 0.3588 | 6.3792 |  | 0.2933 | 2.9609 |
| IL-1B | 0.0095 | 0.1296 |  | -0.0401 | -1.0353 |  | -0.0555 | -0.8136 |
| GDM×IL-1B | -0.0412 | -0.3190 |  | 0.0205 | 0.3027 |  | 0.0270 | 0.2265 |
| *R^2^* | 0.1717 | |  | 0.2261 | |  | 0.0631 | |
| *F* | 10.2946 | |  | 14.5088 | |  | 3.3455 | |
| GDM | 0.7230 | 4.1557 |  | 0.3718 | 4.0483 |  | 0.3648 | 2.2643 |
| IL-6 | -0.0028 | -0.0496 |  | -0.0252 | -0.8540 |  | -0.0367 | -0.7079 |
| GDM×IL-6 | -0.0670 | -0.8815 |  | -0.003 | -0.0080 |  | -0.0274 | -0.3896 |
| *R^2^* | 0.1812 | |  | 0.2282 | |  | 0.0728 | |
| *F* | 10.9914 | |  | 14.6863 | |  | 3.8981 | |
| GDM | 0.5952 | 1.4480 |  | 0.0678 | 0.3128 |  | 0.1201 | 0.3132 |
| IL-10 | 0.0968 | 1.8394 |  | 0.0205 | 0.7383 |  | 0.0531 | 1.0811 |
| GDM×IL-10 | -0.0016 | -0.0144 |  | 0.0824 | 1.4116 |  | 0.0500 | 0.4839 |
| *R^2^* | 0.1945 | |  | 0.2430 | |  | 0.0737 | |
| *F* | 11.9963 | |  | 15.9451 | |  | 3.9501 | |
| GDM | 0.6824 | 3.8793 |  | 0.3103 | 3.3501 |  | 0.2437 | 1.4930 |
| IL-12 | 0.0310 | 0.5194 |  | -0.0314 | -0.9964 |  | -0.0269 | -0.4847 |
| GDM×IL-12 | -0.0728 | -0.6399 |  | 0.0394 | 0.6575 |  | 0.0428 | 0.4057 |
| *R^2^* | 0.1736 | |  | 0.2252 | |  | 0.0601 | |
| *F* | 10.4353 | |  | 14.4328 | |  | 3.1757 | |
| GDM | 0.4934 | 1.5949 |  | 0.1682 | 1.0210 |  | 0.0077 | 0.0265 |
| IL-17A | 0.0623 | 1.1860 |  | -0.0224 | -0.8044 |  | -0.0282 | -0.5764 |
| GDM×IL-17A | 0.0439 | 0.4123 |  | 0.0705 | 1.2513 |  | 0.1062 | 1.0697 |
| *R^2^* | 0.1860 | |  | 0.2282 | |  | 0.0656 | |
| *F* | 11.3463 | |  | 14.6867 | |  | 3.4876 | |
| GDM | 0.8299 | 2.7013 |  | 0.2679 | 1.6481 |  | 0.7625 | 2.6915 |
| TNF-α | 0.0789 | 1.1632 |  | -0.0222 | -0.6176 |  | 0.0784 | 1.2523 |
| GDM×TNF-α | -0.0942 | -0.8503 |  | 0.0373 | 0.6358 |  | -0.1783 | -1.7448 |
| *R^2^* | 0.1787 | |  | 0.2224 | |  | 0.0776 | |
| *F* | 10.8083 | |  | 14.2063 | |  | 4.1782 | |
